# Clock mediates liver senescence by controlling ER stress

**DOI:** 10.18632/aging.101353

**Published:** 2017-12-22

**Authors:** Gongsheng Yuan, Bingxuan Hua, Tingting Cai, Lirong Xu, Ermin Li, Yiqing Huang, Ning Sun, Zuoqin Yan, Chao Lu, Ruizhe Qian

**Affiliations:** ^1^ Department of Physiology and Pathophysiology, School of Basic Medical Sciences, Fudan University, Shanghai 200032, China; ^2^ Shanghai Key Laboratory of Clinical Geriatric Medicine, Research Center on Aging and Medicine, Fudan University, Shanghai 200032, China; ^3^ Department of Orthopedics, Zhongshan Hospital, Fudan University, Shanghai 200032, China; ^4^ State Key Laboratory of Medical Neurobiology, Fudan University, Shanghai 200032, China; ^5^ Department of Gastroenterology, Huadong Hospital, Shanghai Medical College of Fudan University, Shanghai 200040, China

**Keywords:** circadian locomotor output cycles kaput, endoplasmic reticulum stress, ROS, PDIA3, UPR, PERK

## Abstract

Accumulated evidence indicates that circadian genes regulate cell damage and senescence in most mammals. Endoplasmic reticulum (ER) stress and reactive oxygen species (ROS) regulate longevity in many organisms. However, the specific mechanisms of the relationship between the circadian clock and the two stress processes in organisms are poorly understood. Here, we show that Clock-mediated Pdia3 expression is required to sustain reactive oxidative reagents and ER stress. First, ER stress and ROS are strongly activated in the liver tissue of *Clock^Δ19^* mutant mice, which exhibit a significant aging phenotype. Next, transcription of Pdia3 is mediated by the circadian gene Clock, but this process is affected by the *Clock^Δ19^* mutant due to the low affinity of the E-box motif in the promoter. Finally, ablation of Pdia3 with siRNA causes ER stress with sustained phosphorylation of PERK and eIF1α, resulting in exaggerated up-regulation of UPR target genes and increased apoptosis as well as ROS. Moreover, the combined effects result in an imbalance of cell homeostasis and ultimately lead to cell damage and senescence. Taken together, this study identified the circadian gene Clock as a regulator of ER stress and senescence, which will provide a reference for the clinical prevention of aging.

## INTRODUCTION

Aging can be described at molecular, cellular, and organ levels. At the cellular level, aging is accompanied by DNA damage accumulation and loss of genomic integrity [1]. Cell damage, if not fixed, can cause an imbalance of the microenvironment and homeostasis, leading to senescence. Free radicals have been implicated in the mechanism of senescence. Reactive oxygen species (ROS) play an important role in the pathogenesis of diseases of the body. The abnormal increase in ROS lead to an increase in oxidative damage caused by free radicals [2]. In addition to oxidative damage, there are many important quality control mechanisms in cells that can determine cell fate in an adaptive or suicidal manner in response to stress, such as DNA repair, cell growth regulation, and endoplasmic reticulum (ER) stress [3]. The destruction of cell quality control can cause a variety of diseases as well as physical disorders.

At the molecular level, destruction of protein homeostasis is also related to aging. Protein misfolding can lead to residues of hydrophobic amino acids, which cause ER stress [4]. Accumulation of virulence proteins activates the unfolded protein response (UPR) to alleviate the ER stress response. Three transmembrane sensors are involved in the UPR, protein kinase R-like endoplasmic reticulum kinase (PERK), inositol-requiring enzyme-1α (IRE1α), and activating transcription factor 6 (ATF6). The PERK pathway inhibits protein translation, while IRE1α and ATF6 increase expression of ER chaperone proteins and C/EBP homologous protein (CHOP) [5]. However, excessive UPR reactions produce large amounts of reactive oxygen species that cause oxidative stress. There are two sources of ROS in the ER; one is generated by the protein disulfide-isomerase (PDI) electron transfer process, and the other is the impact of mitochondrial production [6].

PDI is a critical ER chaperone that is involved in the folding of catalyzed oxidative proteins and the production of disulfide bonds. For example, PDIA3 is directly involved in disulfide oxidation and isomerization and is central to glycoprotein folding in the ER of mammalian cells [7]. PDIA6 causes ER stress with sustained autophosphorylation of IRE1α and splicing of X-box binding protein 1 (XBP1) mRNA [8]. Many UPR proteins and ER chaperones are closely related to biological rhythms; for example, the biological clock controls the UPR proteins to regulate the ability of ER proteins to meet the metabolic needs of the liver [9].

The circadian rhythm regulates most light-sensitive organisms. Core clock genes rhythmically regulate gene expression and produce biological activity. The biological circadian rhythm has a positive and negative feedback control loop of approximately 24 hours [10]. Two core clock genes, Clock and Bmal1, directly regulate the transcription of target genes by binding to the E-box sequences in the promoter of the output genes [11]. Disorders of biological rhythm can cause physiological function deterioration [12]. The current study found that the human biological Clock gene may be directly involved in human aging and aging-related diseases [13].

In our work, *Clock* mutant mice (*Clock^△19^* mice) exhibited obvious liver aging phenotype. Moreover, the *Clock^△19^* mutant-induced PDIA3 deficiency aggravated ER stress and promoted the production of ROS. Finally, we proved that the imbalance of these factors may be an important cause of liver aging, which may provide a new avenue for the prevention of senescence.

## RESULTS

### *Clock^Δ19^* mice exhibit premature liver aging

**Figure 1:**
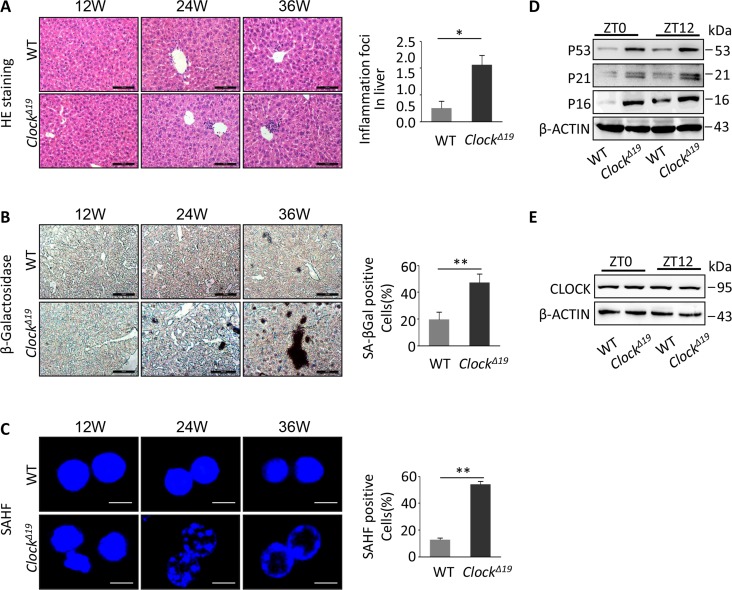
Accelerating the liver aging phenotypes of mice with *Clock^Δ19^* (**A**) Histological analysis of WT and *Clock^Δ19^* mice (12, 24 and 36 week old). HE staining of inflammation foci in liver. Data were analyzed by Student's t-test and displayed as the mean ± S.E.M. Asterisks indicate values significantly different from WT (n=4 for all groups). ^**^, *P*<0.01; ^*^, *P*<0.05. (**B**) Liver tissues stained for SA-β-gal activity. The results are expressed as the mean ± S.E.M. A significant increase in the number of positive areas was observed with *Clock^Δ19^* (n=4 for all groups). ^**^, *P*<0.01; ^*^, *P*<0.05. (**C**) Hepatocytes were stained for SAHF foci in WT and *Clock^Δ19^* mice. Note that the livers of *Clock^Δ19^* mice show a clear aging phenotype. The results of the SAHF analysis are representative images of four experiments. ^**^, *P<0.01*; ^*^, *P<0.05.* (**D**) Immunoblots of liver tissue from WT and *Clock^Δ19^* mice at ZT0 and ZT12 for P53, P21 and P16 indicate the accelerated aging phenotypes. β-ACTIN was used as the loading control. ^**^, *P < 0.01* and ^*^, *P < 0.05* versus control. n=4 mice per group. (**E**) Immunoblots of *Clock^Δ19^* in mouse liver tissue at ZT0 and ZT12. Note that there was no change in the protein levels in *Clock^Δ19^* mice (n=4). ^**^, *P<0.01*; ^*^, *P<0.05*.

To study the potential impact of Clock exon 19 deficiency in mice, we investigated whether the main metabolic organ, the liver, was affected in *Clock^Δ19^* mice. The liver tissue of *Clock^Δ19^* mice displayed higher inflammation activity, higher β-galactosidase activity (an indicator for cellular senescence) and more senescence-associated heterochromatin foci (SAHF)-positive cells than wild-type (WT) tissue in 12 week, 24 week and 36 week age old (Figure 1A-C). Furthermore, we measured the expression of senescent marker proteins to examine the aging of the liver at the molecular level. We found that protein expression of P53, P21 and P16 was significantly higher in *Clock^Δ19^* than WT mice both during the day (ZT0) and night (ZT12) (Figure 1D). Interestingly, Clock protein expression in *Clock^Δ19^* mice was not significantly different between ZT0 or ZT12 (Figure 1E).

### *Clock^Δ19^* mice are more susceptible to oxidative stress and DNA damage

**Figure 2:**
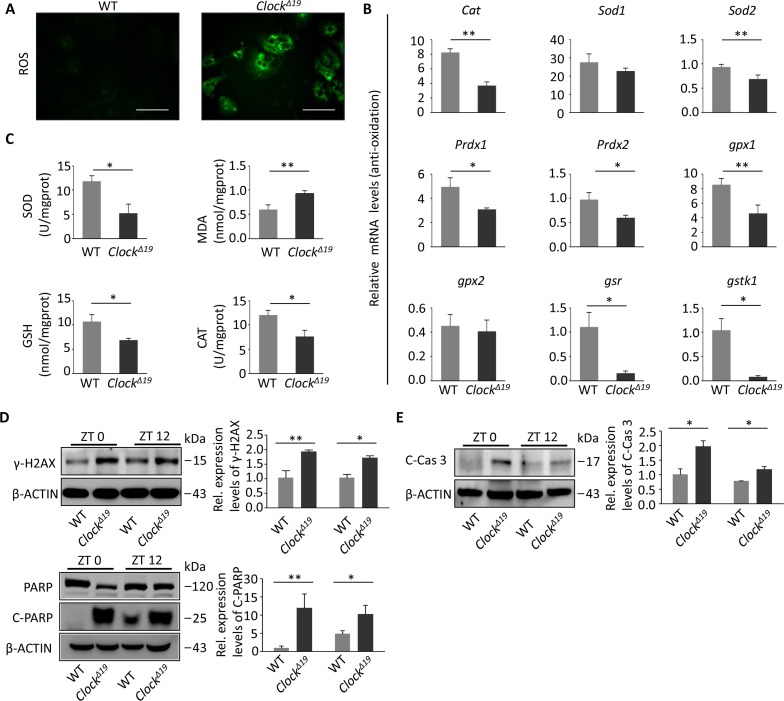
*Clock*^Δ*19*^ mice exhibit oxidative damage and DNA damage (**A**) ROS activities were detected in the primary hepatocytes of WT and *Clock^Δ19^* mice (n=4 for all groups). (**B**) Relative expression of oxidation-related genes in WT and *Clock^Δ19^* mice. Data were analyzed by Student's t-test and displayed as the mean ± S.E.M. (n=4). ^**^, *P<0.01*; ^*^, *P<0.05*. (**C**) SOD, MDA, GSH and CAT activities were analyzed in liver tissue homogenate from WT and *Clock^Δ19^* mice (n=4). ^**^, *P*<0.01; ^*^, *P*<0.05. (**D**) Immunoblots of γ-H2AX and PARP in mouse liver tissues at ZT0 and ZT12. The spliced form of PARP (C-PARP) indicates DNA damage. Note that γ-H2AX was activated in *Clock^Δ19^* mice (n=4). ^**^, *P <* 0.01 and ^*^, *P <* 0.05 versus control. n=4 mice per group. (**E**) Immunoprecipitation of activated Caspase 3 (Cleaved-Caspase 3), which is activated in response to liver cell apoptosis activity. Mice had the same treatment as in (**D**). (n=4). ^**^, *P* < 0.01; ^*^, *P* < 0.05.

To understand why *Clock^Δ19^* would accelerate aging, we investigated the most common causes of aging, i.e., oxidative stress and DNA damage. First, we extracted mouse primary hepatocytes and stained them with dichloro-dihydro-fluorescein diacetate (DCFH-DA) (ROS probe). *Clock^Δ19^* hepatocytes exhibited significantly higher ROS activity than WT cells (Figure 2A). Moreover, we examined the expression of genes associated with oxidative stress and found that most of the antioxidant genes, such as *Cat*, *Sod2*, *Prdx1*, *Prdx2*, *Gpx1*, *Gpx2*, *Gsr* and *Gstk1*, were significantly down-regulated, reflecting changes in the degree of oxidation in *Clock^Δ19^* hepatocytes at the genetic level (Figure 2B). Then, we detected the activity of oxidative stress-related proteins in the mouse liver homogenate, and the activity of antioxidant indexes, such as superoxide dismutase (SOD), glutathione (GSH), and catalase (CAT), was significantly lower, while the activity of the oxidative index malondialdehyde (MDA) was significantly higher than in the WT homogenate (Figure 2C). Oxidative damage is often accompanied by severe genetic damage. Therefore, our next step was to detect the expression levels of proteins that react to DNA damage [γ-H2A histone family, member X (γ-H2AX)] and DNA damage repair [poly (ADP-ribose) polymerase (PARP)]. The protein levels of γ-H2AX and cleaved PARP (C-PARP) were significantly elevated in the liver of *Clock^Δ19^* mice at ZT0 and ZT12 (Figure 2D), suggesting that the liver was significantly damaged. Since both oxidative stress and DNA damage are associated with apoptosis and Caspase 3 is involved in the activation of apoptotic pathways, we found that activation of Caspases 3 was also significantly increased in *Clock^Δ19^* mice (Figure 2E).

### *Clock^Δ19^* induces significant ER stress and the unfolded protein response

**Figure 3:**
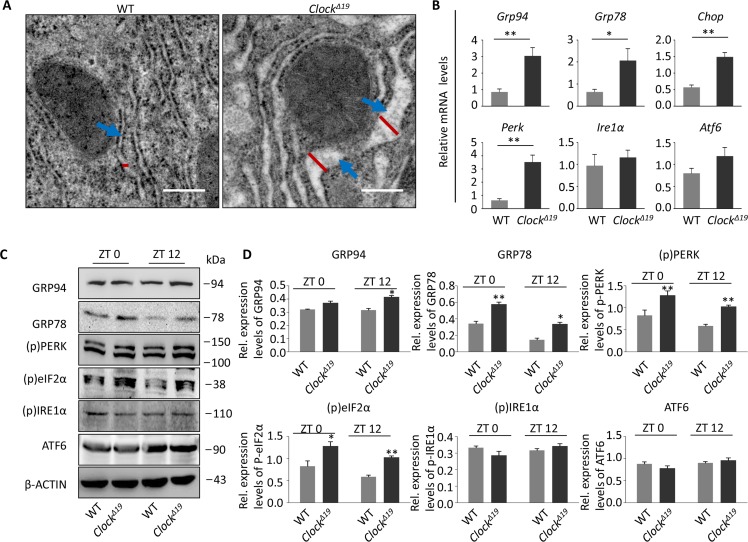
*Clock^Δ19^* results in ER stress in mice (**A**) TEM depicting the luminal diameter of the ER in WT and *Clock^Δ19^* hepatocytes. *Clock^Δ19^* mouse hepatocytes show the expansion of the ER (blue arrow; n=4 for all groups). (**B**) Relative expression assessed by qPCR of the UPR pathway genes in the liver of WT and *Clock^Δ19^* mice from 16 weeks of age. Note that the expression of *Grp94*, *Grp78*, *Chop* and *Perk* were significantly increased in *Clock^Δ19^* mice. Data were normalized to *Gapdh* expression (n=4). ^**^, *P <* 0.01 and ^*^, *P <* 0.05. (**C**-**D**) Immunoblots of liver tissue from WT and *Clock^Δ19^* mice at ZT0 and ZT12; the UPR proteins GRP94, GRP78, (p)PERK and (p)eIF2α are significantly activated in *Clock^Δ19^* mice. β-ACTIN is a loading control. ^**^, *P <* 0.01 and ^*^, *P <* 0.05 versus control. n=4 mice per group.

Interestingly, Dmitri V. Krysko et al. argued that high ROS could damage the protein folding ability and capacity of the ER, thereby interfering with the ER steady state [14]. We wondered whether ER stress was present in *Clock^Δ19^* mice. First, through transmission electron microscopy, we found a significant expansion of the ER (blue arrow: ER; red line: ER diameter) in *Clock^Δ19^* hepatocytes (Figure 3A), suggestive of severe ER stress.

Interestingly, in response to stress, the ER activates a series of complex signaling pathways that are a part of the UPR. Here, we measured the expression of several major UPR pathway genes and found that most of the UPR-related genes (*Grp94*, *Grp78*, *Chop*, *Perk*, *Ire1α* and *Atf6*), especially *Perk*, were significantly up-regulated, whereas *Ire1α* and *Atf6* were only minorly up-regulated compared to the expression in the WT liver (Figure 3B). Moreover, the expression of most of the UPR proteins was either significantly up-regulated at ZT0 or at ZT12 (Figure 3C-D). In contrast to the gene expression results, immunoblots showed that the UPR response was mainly activated by the PERK path-way, as there was a significant increase in phosphorylation-activated (p)PERK and its downstream phosphorylated eukaryotic translation initiation factor 2 alpha ((p)eIF2α) but not phosphorylated IRE1α and ATF6 in *Clock^Δ19^* compared to that in WT mice (Figure 3C-D).

### *Clock^Δ19^* mice exhibit deficient expression of PDIA3

Next, we demonstrated that expression of most of the UPR genes showed a circadian rhythm in the mouse liver from ZT0 to ZT12 (Supplementary Figure 1), suggesting that these UPR genes may be controlled by the biological clock.

**Figure 4:**
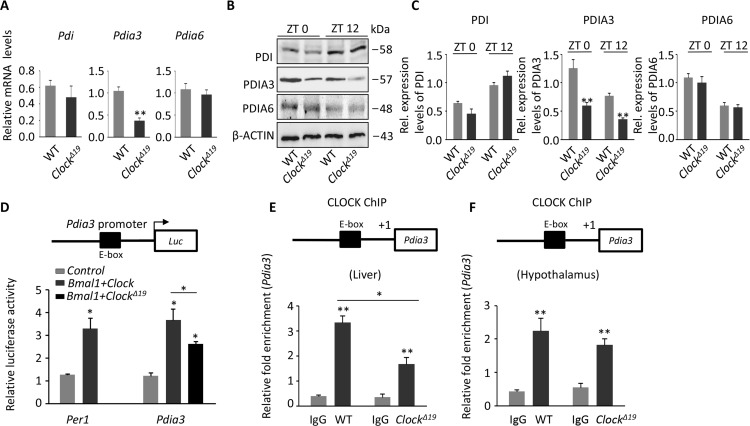
The loss of PDIA3 shows in *Clock^Δ19^* mice (**A**) Relative expression assessed by qPCR of the PDI family genes (*Pdi*, *Pdia3*, and *Pdia6*). Note that the PDI family genes, especially *Pdia3*, were down-regulated in *Clock^Δ19^* mice. RNA was collected from WT and *Clock^Δ19^* mice at ZT0 and ZT12 (n=4). Data were normalized to *Gapdh* expression. ^**^, *P <* 0.01 and ^*^, *P <* 0.05 versus control. (**B**-**C**) Immunoblots of PDI family proteins (PDI, PDIA3, and PDIA6) at ZT0 and ZT12 from WT and *Clock^Δ19^* mice. Quantification of the immunoblots show significantly reduced expression of PDIA3. ^**^, *P <* 0.01 and ^*^, *P <* 0.05 versus control. n=4 mice per group. (**D**) The dual luciferase method shows that BMAL1:CLOCK can regulate Pdia3 at the transcriptional level. Per1, which is activated by BMAL1:CLOCK, was used as a positive control. HEK293T cells were co-transfected with *Pdia3-luc* and *Bmal1:Clock* or *Bmal1:Clock^Δ19^*, and the results are expressed as the firefly luciferase activity normalized to renilla luciferase activity. Note that overexpression of BMAL1:CLOCK (dark gray bar) transactivated the *Per1* and *Pdia3* reporter genes more than in the control transfections (light gray bar). Activation of the Pdia3 reporter was significantly decreased by 30% when *Clock^Δ19^* was overexpressed with BMAL1 (black bar). The results are expressed as the mean ± S.E.M (n=4). ^**^, *P <* 0.01 and ^*^, *P <* 0.05. (**E**-**F**) ChIP assay showing the binding of CLOCK to the E-box (CACGTG) of *Pdia3* in liver and hypothalamus tissue. Error bars show the S.E.M of the ChIP PCR reactions performed in triplicate (n=4). ^**^, *P*<0.01; ^*^, *P*<0.05.

Then, we analyzed the raw microarray gene expression data (GEO: GSE454), obtained from WT and *Clock^Δ19^* mouse livers. Interestingly, expression of some PDI-related genes (*Pdi*, *Pdia3* and *Pdia6*), which exist in the ER lumen and provide a disulfide bond, was changed significantly. Thus, we experimentally validated the changes in the PDI-related gene and protein levels. Surprisingly, the gene and protein levels of the PDI-related genes (PDI, PDIA3, and PDIA6) were reduced, with the reduction in Pdia3 the most pronounced (Figure 4A-C).

Transcription factors of the biological clock regulate the expression of most of the genes that are expressed in a circadian rhythm. We wanted to know whether *Pdia3* was also directly regulated by the transcription of the Clock gene. Using Jessica L's method [15], we constructed mouse *Bmal1:Clock* or *Bmal1:Clock^Δ19^* vectors and the *Pdia3* luciferase vector and co-transfected them into 293T cells (Figure 4D). Here, both BMAL1:CLOCK and BMAL1:CLOCK^Δ19^ significantly activated the transcription of *Pdia3*, but the activation effect of BMAL1:CLOCK^Δ19^ was lower (Figure 4D). Clock played a transcriptional role mainly by binding to the promoter region of the target gene on the E-box (CACGTG). Therefore, chromatin immunoprecipitation (ChIP) was used to confirm the binding of CLOCK to the E-box (*Pdia3* promoter) in the WT and *Clock^Δ19^* mouse liver (Figure 4E) and hypothalamus tissue (Figure 4F). The results indicated that CLOCK could bind the E-box structure of the *Pdia3* promoter, but the binding capacity in *Clock^Δ19^* mice was significantly reduced (Figure 4E-F).

### Loss of PDIA3 activates the PERK but not the IRE1α or ATF6 pathway

**Figure 5:**
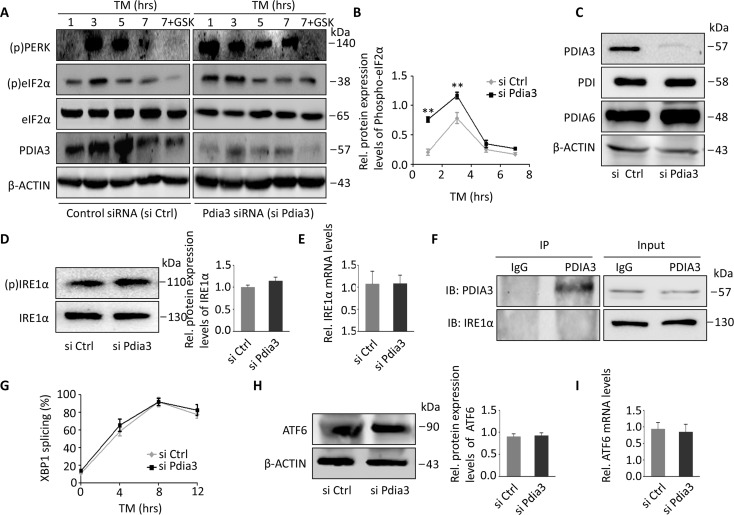
The lack of Pdia3 leads to activation of PERK but not IRE or ATF6 (**A**-**B**) Control siRNA (siCtrl)- and Pdia3 siRNA (siPdia3)-treated AML12 cells were exposed to 2 μg/mL TM for the indicated times, and GSK2606414 (PERK inhibitor) was introduced with TM treatment at the indicated concentration for 7 hours. Cell lysates were then immunoblotted to detect (p)PERK, (p)eIF2α, eIF2α and PDIA3. In addition, the mean ± S.E.M. of the phosphorylated /total eIF2a ratio (normalized to eIF2a) is shown in (B) (n=4). ^**^, *P*<0.01; ^*^, *P*<0.05. (**C**) Immunoblotting of cells from siCtrl- and siPdia3-treated AML12 cells to show the level of PDIA3 depletion. Note that no change was found in levels of PDI or PDIA6 (n=4). (**D**-**E**) Immunoblots (**D**) and relative mRNA expression (**E**) of IRE1*α* in siCtrl- and siPdia3-treated AML12 cells. No significant difference was found. (n=4); ^**^, *P*<0.01; ^*^, *P*<0.05. (**F**) Endogenous immunoprecipitation of PDIA3 pulled down endogenous IRE1α from HEK293T cell lysates; 1% of lysate was used as the input control (n=3 experiments). (**G**) siCtrl- or siPdia3-treated AML12 cells were incubated with 2 μg/mL TM for the indicated times, and un-spliced (u) and spliced (s) XBP1 mRNA was amplified by qPCR. *Gapdh* served as the control. Means ± S.E.M are plotted. ^**^, *P < 0.01* and ^*^, *P < 0.05* versus control. n=4 mice per group. (**H**-**I**) Immunoblots (**H**) and relative mRNA expression (**I**) of ATF6 in siCtrl- and siPdia3-treated AML12 cells. (n=4) ^**^, P < 0.01 and ^*^, P < 0.05 versus control.

Furthermore, we used siRNA to interfere with *Pdia3* expression in mouse AML12 hepatocytes to verify whether a reduction in Pdia3 was the main cause of the UPR. The previous experiment suggested that the PERK pathway of *Clock^Δ19^* mice was the most active UPR pathway (Figure 3B-D). Therefore, we first tested the activity of the PERK pathway at different time points (1, 3, 5, 7 h) and used a PERK inhibitor, GSK2606414 (GSK), to observe the changes in PERK under the continuing stress effect of tunicamycin (TM, an ER stress inducer). We found that the levels of (p)PERK were highest in the first hour in cells treated with siRNA against *Pdia3*, while in the control group, PERK was activated at the third hour. Then, the (p)PERK levels in the two groups were gradually attenuated (Figure 5A). In contrast, GSK completely inhibited the phosphorylation of PERK in these two groups (Figure 5A). (p)eIF2α was the downstream target of PERK activation, and its expression level was significantly higher in cells with *Pdia3* inhibition and TM than in the control group, especially at the first and third hour (Figure 5B). PDI or PDIA6 have a known role in the folding of glycoproteins that may have an important impact on ER stress, but the ablation of the PDIA3 protein did not affect the expression of PDI or PDIA6 (Figure 5C).

Next, we needed to determine whether IRE1α gene expression and phosphorylated IRE1α levels were altered by inhibition of Pdia3 in TM-treated AML12 hepatocytes. The levels of (p)IRE1α were only slightly but not significantly higher in these cells, and gene expression was not significantly changed (Figure 5D-E). Davide Eletto et al. suggested that PDIA6 affected the UPR reaction by binding and activating IRE1α [8]. As a homologous analogue of PDIA6, we wondered whether PDIA3 could also bind to IRE1α. However, immunoprecipitation showed that PDIA3 did not bind to IRE1α (Figure 5F). Additionally, IRE1α has been reported to splice and thereby activate XBP1, but our results showed that the level of spliced XBP1 did not change significantly (Figure 5G). We also detected the expression of the ATF6 gene and protein in the same way with constant TM administration and inhibition of *Pdia3*, but neither the gene nor the protein levels changed significantly (Figure 5H-I). Thus, we hypothesized that Pdia3 might not function through the IRE1α or ATF6 pathway.

### Knockdown of PDIA3 induces oxidative stress

Recent reports have shown that continuous ER stress can cause ROS [16,17], suggesting a cross-talk between the two. We sought to determine if the suppression of Pdia3, serving as a cross-talk regulator, could stimulate ROS production by inducing ER stress.

**Figure 6:**
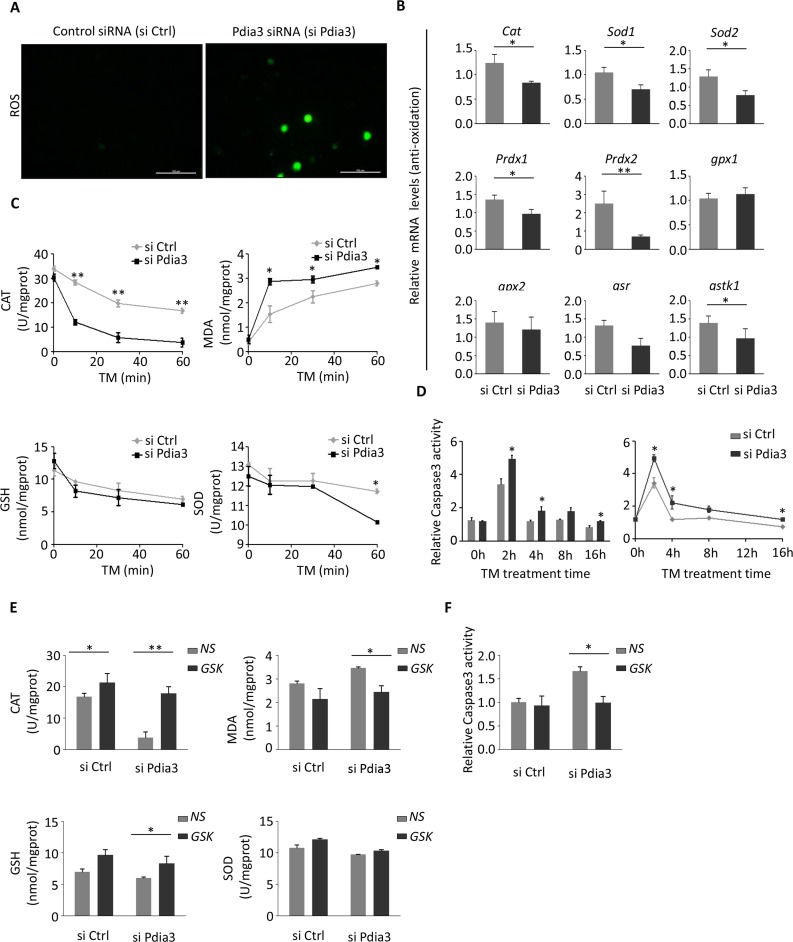
Loss of PDIA3 promotes oxidative stress and apoptosis via the PERK pathway (**A**) ROS activities were detected in the control siRNA (siCtrl)-treated and Pdia3 siRNA (siPdia3)-treated AML12 cells. After transfection for 24 hours, cells were exposed to 2 μg/mL TM for 6 hours; n=4 for all groups. (**B**) Relative Caspase 3 activity were detected in TM-treated AML12 cells. The cell treatment method was the same as in A. Caspase activity was measured in the cell lysate after addition of TM. The left panel shows the activity of the caspases after Pdia3 inhibition at a specific time point. The panel on the right shows the corresponding line graph. (n=3). ^**^, *P*<0.01; ^*^, *P*<0.05. (**C**) SOD, MDA, GSH and CAT activities were analyzed in AML12 cell homogenate treated as in (A) at the indicated times (0, 20, 40, and 60 minutes). CAT, GSH and SOD represent the antioxidation level; MDA represents the oxidation level. (n=4) ^**^, *P*<0.01; ^*^, *P*<0.05. (**D**) Relative expression assessed by qPCR of antioxidation genes (*Cat, Sod, Sod2, Prdx1, Prdx2, gpx1, gpx2, gsr,* and *gstk1*). Light gray represents the control group (siCtrl); dark gray represents Pdia3-inhibited group (siPdia3). Data were normalized to *Gapdh* expression. (n=4) ^**^, *P* < 0.01 and ^*^, *P* < 0.05 versus control. (**E**) AML12 cells were treated with the GSK inhibitor during the TM stress (2 μg/mL). Cell lysates were analyzed by the method used in (**C**) (n=4). ^**^, *P*<0.01; ^*^, *P*<0.05. (**F**) Relative activity of Caspase 3 was analyzed with the PERK inhibitor (GSK) and TM stress (2 μg/mL). (n=4) ^**^, *P* < 0.01 and ^*^, *P* < 0.05 versus control.

First, we measured the ROS level of AML12 hepatocytes after 6 hours of TM treatment and inhibition of *Pdia3* (Figure 6A). The ROS levels were significantly increased after *Pdia3* inhibition, suggesting that ROS production might be regulated by *Pdia3* (Figure 6A). Then, we further determined if the expression of oxidative stress-related genes would also change at the molecular level. The results indicated that expression of most of the genes involved in antioxidation (*Cat, Sod1, Sod2, Prdx1, Prdx2* and *Gstk1*) was significantly reduced after administration of TM and Pdia3 inhibition, but the reduction in *Gpx1, Gpx2* and *Gsr* was not significant (Figure 6B).

To determine how the protein activity of cells involved in oxidative stress at different time points was changed by TM stimulation and *Pdia3* inhibition, we measured the oxidative and antioxidant levels of cell lysates directly. The activity of CAT, GSH and SOD decreased in response to the reduction in cell antioxidant levels over time in both the Pdia3 siRNA (siPdia3)- and control siRNA (siCtrl)-treated groups (Figure 6C). After Pdia3 inhibition, CAT activity was significantly lower than in the control group at 10 minutes, and SOD activity was lower than in the control group at 60 minutes. However, the GSH content did not change in these two groups over time (Figure 6C). For oxidative levels, MDA activity was significantly higher in the first 10 minutes in the siPdia3-treated group than in the control group and continued to increase over time (Figure 6C).

Oxidative stress usually leads to apoptosis. Thus, we examined the activation of Caspase 3 (Figure 6D) at different time points. The results showed that apoptosis was significantly activated in both the control group and the Pdia3 inhibition group after TM administration, but loss of Pdia3 caused a more significant increase in the activation of these apoptosis-related proteins (Figure 6D). With continued TM stimulation, the levels of activated Caspase 3 increased significantly after the first 2 hours but then decreased with the passage of time, and the difference between the control group and the siPdia3-treated group was also reduced (Figure 6D).

In this stage of the UPR, a large number of ROS, such as H_2_O_2_, O_2_·^−^ and ·OH, are produced [6,18] Thus, we hypothesized that inhibition of Pdia3 could cause a severe ER stress response and mainly activate the PERK pathway of the UPR, leading to ROS activation. Therefore, we used GSK2606414 to inhibit the PERK pathway to see if there was a change in oxidative stress. The results showed that the antioxidant activity of CAT, GSH and SOD was increased, especially that of CAT and GSH and the activity of MDA was significantly decreased by GSK inhibition under the combined action of TM and inhibited PDIA3 (Figure 6E). Moreover, the level of apoptosis activation induced by siPdia3 was also significantly reduced after GSK inhibition (Figure 6F).

### Pdia3 can relieve the aging phenotype through inhibition of the PERK pathway

**Figure 7:**
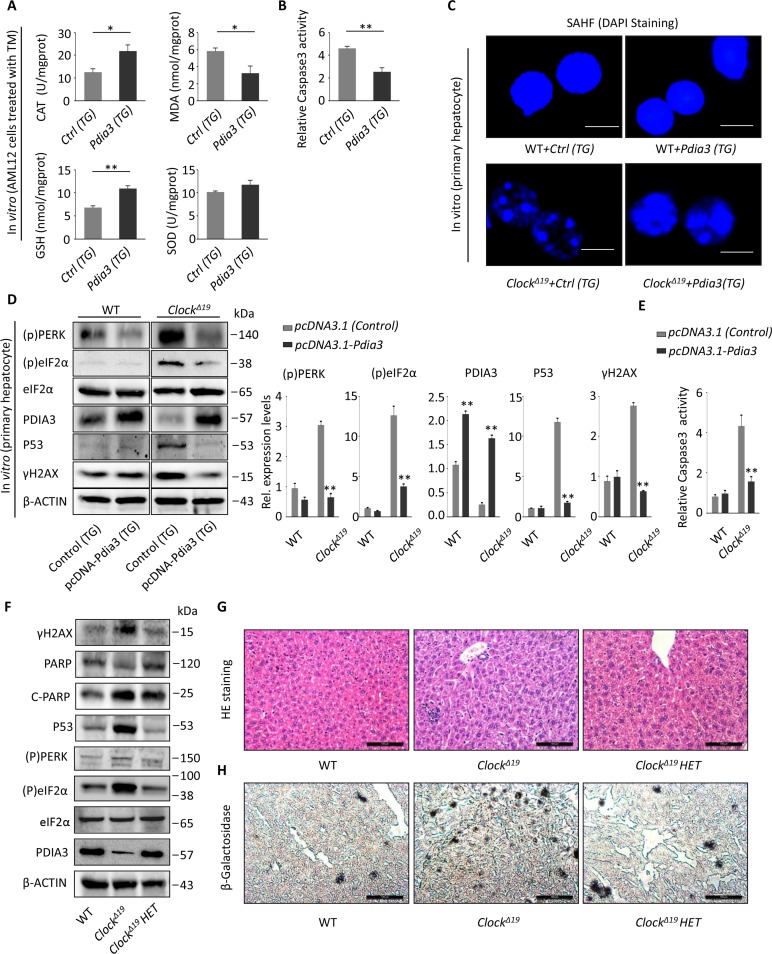
Pdia3 can reverse the aging process of the liver in *Clock^Δ19^* mice (**A**) AML12 cells were transfected with the pcDNA3.1-Pdia3 plasmid (Trans gene, TG) for 24 hours and then stimulated with TM stress (2 μg/mL) for 2 hours. SOD, MDA, GSH and CAT activity were analyzed in the cell homogenate. (n=4) ^**^, *P*<0.01; ^*^, *P*<0.05. (**B**) AML12 cells were treated as described in (**A**), and the activity of Caspase 3 was measured to reflect apoptotic activity. (n=4) ^**^, P < 0.01 and ^*^, P < 0.05 versus control. **(C**) SAHF stained via DAPI were visualized by fluorescence microscopy. The primary hepatocytes were extracted, and the number of SAHF was observed after transfection with the pcDNA3.1-Pdia3 plasmid for 24 hours. The SAHF were greatly rescued in *Clock^Δ19^+Pdia3(TG)* cells (n=3). (**D**) The primary liver cells were extracted and transfected with the pcDNA3.1-Pdia3 plasmid for 48 hours. Immunoblotting methods were used to detect the expression of UPR proteins (p)PERK and (p)eIF2α, PDIA3, senescent protein P53, and DNA damage protein γ-H2AX. The statistical results are displayed (right). The results are expressed as the mean ± S.E.M (n=4). ^**^, *P <* 0.01 and ^*^, *P <* 0.05. (**E**) Primary liver cells were extracted and treated as described in (**D**). Then, the relative activity of Caspase 3 was detected to reflect the level of apoptosis. Note that the apoptotic activity was significantly reduced after transfection with Pdia3. (n=4) ^**^, *P*<0.01; ^*^, *P*<0.05. (**F**) Immunoblots showing the expression levels of γH2AX, PARP, P53, (p)PERK, (p)eIF2a and PDIA3 in the WT, *Clock^Δ19^* and *Clock^Δ19^* heterozygote groups (36 week old). n=3 for all groups. (**G**-**H)** Histological analysis of mice as described in (**F**). HE and SA-β-gal staining of the liver tissue. (n=3 for all groups). Note that the *Clock^Δ19^ HET* mice show a decreased inflammation foci and improved aging phenotype.

Our next question was whether the oxidative stress or apoptotic state could be changed by overexpression of *Pdia3* in TM-stimulated AML12 cells. Forced expression of *Pdia3* resulted in enhanced antioxidant activity, especially activity of CAT and GSH, and decreased oxidative activity of MDA (Figure 7A). Moreover, overexpression of *Pdia3* also significantly reduced the level of activation of apoptotic proteins induced by TM (Figure 7B). Next, we needed to determine whether overexpression of Pdia3 reversed the aging, cell damage, ER stress and apoptotic response caused by *Clock^Δ19^*. Here, we cultured primary liver cells from WT and *Clock^Δ19^* mice and *Clock^Δ19^* mouse liver cells with overexpression of *Pdia3*. First, immunofluorescence staining showed a significant reduction in the number of SAHF after overexpression of *Pdia3* (Figure 7C), suggesting an improvement in cell senescence. Western blot analysis further confirmed that overexpression of *Pdia3* inhibited the PERK pathway, resulting in a decrease in phosphorylation activation of PERK and eIF2α (Figure 7D). Overexpression of Pdia3 also inhibited P53 and γ-H2AX expression (Figure 7D) and reduced the degree of activation of apoptosis (Figure 7E). To further validate the effect of Pdia3 on liver senescence, *Clock^Δ19^* heterozygous mice were examined as another way of restoring Pdia3. First, we investigated whether *Clock^Δ19^* heterozygotes exhibited less cell damage and aging. *Clock^Δ19^* heterozygous mice exhibited reduced γ-H2AX and C-PARP levels (Figure 7F). What is more, the aging process was also alleviated, as indicated by the expression of the senescent marker, P53 (Figure 7F), suggesting that the increase in Pdia3 could alleviate the cell damage and aging caused by *Clock^Δ19^*. Then, we discovered that activation of the PERK pathway of the UPR was significantly inhibited in *Clock^Δ19^* heterozygous mice (Figure 7H), as indicated by the significant reduction in phosphorylation-activated PERK and eIF2α (Figure 7F). Moreover, histological analysis of liver tissue also showed alleviation of the aging phenotype in heterozygous mice (Figure 7G-H).

## DISCUSSION

**Figure 8:**
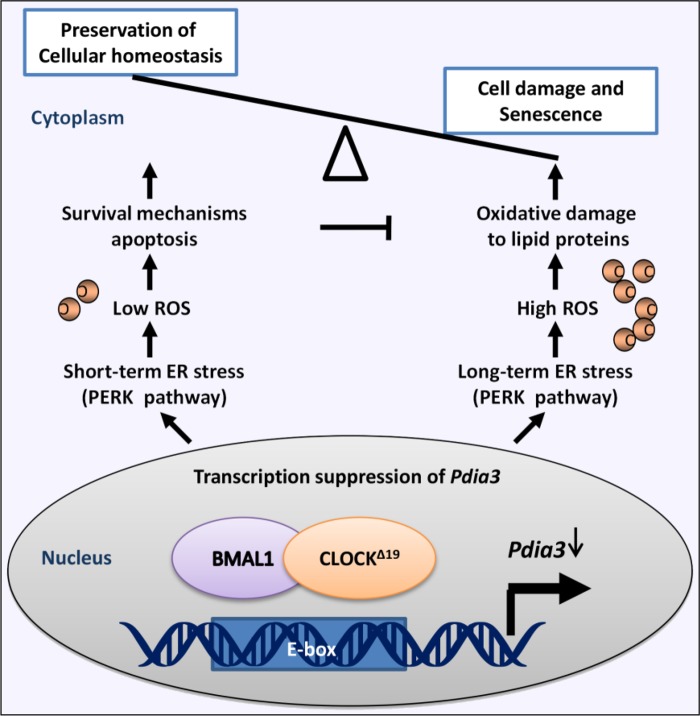
Schematic diagram for the mechanism by which the Clock mutant causes senescence

In this study, the effects of *Clock^Δ19^* on liver senescence were confirmed (Figure 8). First, we found that *Clock^Δ19^* mutant mice showed a significant phenotype of liver aging due to a significant increase in peroxidation and ER stress. Then, *Clock^Δ19^* mutant mice showed reduced levels of Pdia3, which was transcriptionally regulated by BMAL1:CLOCK through binding to the E-box motif. Interestingly, we found that inhibition of *Pdia3* not only led to the activation of the PERK pathway but not IRE1α or ATF6 but also caused activation of oxidative stress and transient apoptosis. Finally, we found that overexpression of *Pdia3* could inhibit ER stress, oxidative damage, and the aging phenotype via the PERK pathway of the UPR.

Interference with the biological clock results in significant senescence. Recent reports have shown that knockout of Clock leads to cataracts and a 15% reduction in life expectancy [19]. However, neural PAS domain protein 2 (NPAS2) compensates for the loss of Clock to maintain the rhythm in the brain. However, the *Clock^Δ19^* mutant combines with Bmal1 to form a dimer and cause a rhythm disorder, resulting in a more pronounced metabolic disorder in *Clock^Δ19^* mice than in Clock knockout mice [20]. Moreover, Antoch MP et al. confirmed that *Clock^Δ19^* mice have a decreased lifespan and a corresponding senescence phenotype under certain conditions [21]. Here, for the first time, we confirmed that liver aging is present in *Clock^Δ19^* mice. Metabolic syndrome is the most obvious phenotype of *Clock^Δ19^* mice and may mainly be attributed to the dysfunction of liver compensatory mechanisms [22,23]. In addition, Carlos Lopez-Otin suggested that metabolic disorders may be an important cause of aging because abnormal feeding will lead to a shortened lifespan, and dietary restriction elongate the lifespan [24,25]. Thus, the liver has become an important metabolic organ for our study of aging.

To elucidate the mechanisms of liver senescence, we analyzed the gene expression data from the GEO database (GEO: GSE454) and found that the expression of both oxidative stress genes and ER stress genes was significantly changed. Reports have shown that reactive oxygen species (ROS) are potent inducers of cell damage and aging [2]. In addition, aging and aging-related diseases are related to impaired protein homeostasis or proteostasis [26]. Furthermore, many misfolded proteins lead to the development of some age-related pathologies, such as Alzheimer's disease, Parkinson's disease and cataracts [26]. Here, the liver tissue of *Clock^Δ19^* mice exhibited both significant levels of ROS and increased ER stress. Therefore, we believe that the genetic damage caused by oxidative stress and protein instability in *Clock^Δ19^* mice may be the main causes of aging.

Studies have confirmed that the circadian clock directly controls ROS and ER stress [27]. Numerous circadian-controlled genes have been found to directly or indirectly mediate ROS levels [28]. Recent reports have shown that Clock protects against liver damage by regulating the UPR [9]. Moreover, circadian control of cytoplasmic polyadenylation element binding-protein 4 (CPEB4) regulates a translational response that counteracts hepatic steatosis under ER stress [29]. In our work, most of the UPR genes were significantly up-regulated in *Clock^Δ19^* mice, whereas Pdia3 expression of the UPR was significantly reduced. Thus, we speculated that *Clock*^Δ19^ caused Pdia3 deficiency, which might initiate the UPR. Finally, Pdia3 was confirmed to be transcriptionally regulated by *Bmal1:Clock* and *Bmal1:Clock^Δ19^*, but the regulatory ability of *Bmal1:Clock^Δ19^* was much lower. Nobuya Koike clarified the transcriptional architecture and chromatin landscape of the biological clock by using ChIP sequencing [30]. We analyzed this motif data and found that Pdia3 contained an E-box structure to which BMAL1:CLOCK can bind. We further demonstrated the binding capacity of BMAL1:CLOCK to the E-box structure of the Pdia3 promoter region in the liver and hypothalamus of mice and found a significant reduction in the BMAL1:CLOCK^Δ19^ binding capacity in the liver but not the hypothalamus. These results indicated that peripheral Clock may play a major regulatory role for Pdia3. Similarly, Shimomura K et al. demonstrated that *Clock^Δ19^* mice exhibited a decrease in the affinity of the target gene E-box [31]. We speculate that exon 19 might be an important functional region that could regulate the transcriptional activity of peripheral Clock. Our future experiments will focus on the effect of exon 19 on transcription.

The PERK pathway is one of the main pathways of the UPR, which can activate eIF2α to further promote the production of ATF4. ATF4 is a central transcription factor of the PERK pathway and can be transcriptionally regulated by CLOCK [32], indicating that CLOCK can directly or indirectly affect the PERK pathway. Our results confirmed that only the PERK pathway was activated in *Clock^Δ19^* mutant mice. Moreover, the ablation of *Pdia3* activated the PERK pathway but not IRE1α or ATF6. Therefore, we believe that PERK may be the main pathway regulated by the circadian gene Clock. However, the UPR is a complex process; we cannot completely rule out the role of IRE1α or ATF6 in the UPR.

PDIA3 is a disulfide isomerase as well as an important protein in the UPR that can not only perform protein quality control but also balance the level of ROS[33]. Similarly, in our experiments, inhibition of Pdia3 was shown to significantly promote ROS. Not only that, we also verified that Pdia3 was a key mediator regulating ER stress and ROS crosstalk. That is, the loss of Pdia3 activated peroxidation via the PERK pathway of the UPR. Apoptosis results from ER stress and ROS stimulation and can alleviate aging. Our results show that *Clock^Δ19^* mice exhibit higher apoptotic activity. However, in TM sustained stimulation and Pdia3 inhibition experiments, high apoptotic activity only occurred at the initial stage of ER stress (short-term ER stress), and the activity was significantly reduced over time. Therefore, apoptosis cannot alleviate the cell damage caused by ROS and ER stress. Therefore, we hypothesized that there was persistent ER stress (long-term ER stress) and peroxidation in *Clock^Δ19^* mice, and the apoptotic response could not counteract the cell damage, resulting in aging. Using in vitro and in vivo experiments, we further demonstrated that over-expression of PDIA3 reversed the ER stress, oxidative stress, and senescence caused by *Clock^Δ19^*.

In summary, we conclude that *Clock^Δ19^* induces inhibition of Pdia3, which is the leading cause of liver aging. More importantly, our results report that Clock regulates senescence, providing a medical avenue for preventing senescence.

## MATERIALS AND METHODS

### Animals

Male and female *Clock^Δ19^* and C57/BL6 mice were obtained from the Model Animal Research Center of Nanjing University. All of the animal experiments were approved by the appropriate institutional animal care and use committee. Mice were housed under 12-hour light/12-hour dark cycles for more than 2 weeks in a standard animal facility under controlled temperature and humidity. Each group of mice were fasted for 12 hours before surgery, every 4 hours, in a 24-hour cycle. Mice were anesthetized with 4% chloral hydrate, and liver tissue was snap-frozen in liquid nitrogen and stored at −80°C.

### Plasmid construction

The *Pdia3* reporter plasmid (*Pdia3-luc*), containing the 5′-flanking sequence (2 kilobases) of the *Pdia3* gene, was constructed by ligating the NHeI-HindIII fragment into the pGL3 basic vector (from Promega, Madison, WI). Cloning of the *Clock* or *Pdia3* gene [open reading frame (ORF)] into *pcDNA 3.1* (generating NotI and Apa1 sites) and the Bmal1 gene (OFR) into *pCMV* (generating BamHI and EcoRI sites) was performed by incorporating the appropriate restriction sites at both ends of the gene through amplification, followed by digestion and ligation with the plasmid and transformation. The positive transformants that grew on ampicillin agar plates were sub-cultured. The existence of the *Clock, Bmal1* and *Pdia3* genes was confirmed by PCR and sequencing following plasmid extraction.

### Dual luciferase reporter assay

HEK293T cells in 6-well plates were transiently transfected with 1 μg of *pGL3-Pdia3* plasmid, 1 μg of *pRL-TK* plasmid, and 2 μg of *Bmal1:Clock* or *Bmal1:Clock^Δ19^* plasmid using Lipofectamine^TM^ 2000 reagent (Invitrogen, USA). Cells were harvested 48 hours after transfection and assayed for firefly and renilla luciferase activity using the Dual Luciferase Reporter Gene Assay Kit (Beyotime, China). Firefly luciferase activity was normalized to renilla luciferase activity.

### Cell culture

The murine hepatocyte AML12 cell line (ATCC CRL-2254) was cultured in a 1:1 mixture of Dulbecco's modified Eagle's medium and Ham's F12 medium with an insulin-transferrin-selenium mixture and 40 ng/ml dexamethasone and 10% FBS. (Gibco, life technologies) and incubated at 37°C under 5% CO_2_. Cells were grown in 6-well plates according to the instructions. Lipofectamine^TM^ 3000 (Invitrogen, USA) was used to transfect cells with 3 μg of plasmid for 24 hours.

### Knockdown of gene expression using small interfering RNA (siRNA)

AML12 cells were transfected with siRNA targeting *Pdia3* (1# sense strand: 5′GGACAAGACUGUGGCAUAU 3′ and #2 sense strand: 5′GGGCAAGGACUUACUUAUU 3′) using Lipofectamine^TM^ 3000 reagent (Invitrogen, Paisley, UK) according to the manufacturer's protocol. A final concentration of 100 nM siRNA was used. After transfection for 48 hours, western blot analysis was used to assess the protein content.

### Measurements of oxidant and apoptosis activity

The level of intracellular ROS was quantified using the Reactive Oxygen Species Assay Kit (Beyotime, China). Primary hepatocytes were obtained as described [1], and the cells were washed three times with PBS. DCFH-DA (10 μM) was added, and the cells were incubated for 30 minutes at 37°C in the dark. After the cells were washed three times with PBS, photomicrographs were obtained using an Olympus BX41 microscope (Center Valley, PA) equipped with an Olympus Q-color5 digital camera. The CAT, MDA, GSH, and SOD activities of the liver tissue were measured using commercial kits according to the manufacturer's protocols (Jiancheng Bioengineering Ltd., Nanjing, China). Levels of activated Caspase 3, which reflected apoptosis activity, were detected by using commercial kits (Beyotime, China).

### Transmission electron microscopy (TEM)

Livers from mice were fixed overnight and processed as previously described [2](Verfaillie *et al*. 2012). Nanometer sections were cut and stained with 0.3% (w/v) lead citrate, and images were analyzed at the Electron Microscopy Core Facility (Philips Research, Eindhoven, The Netherlands).

### Quantitative PCR (qPCR)

Tissues or cells were washed with ice-cold PBS, and the total RNA was extracted using TRIzol reagent (Invitrogen, USA). The quality of the extracted RNA was measured in terms of the OD_260_/OD_280_ ratio ranging from 1.9 to 2.1. The cDNA was obtained using a reverse transcription kit according to the manufacturer's instructions (TOYOBO, Japan). qPCR was performed with a Bio-Rad real-time analyzer (Bio-Rad, Hercules, CA). PCR amplification was carried out for a total of 40 cycles and normalized to *Gapdh* expression. All reactions were performed in triplicate, and the relative expression was determined using the 2^−ΔΔCT^ method. The primers used are listed in Table S1(Supporting information).

### Histological examination

Tissue was fixed in 4% paraformaldehyde for a minimum of 48 hours, paraffin-embedded and routinely processed for histology. Then, 5-μm paraffin serial sections were stained with Hematoxylin-Eosin (HE).

### Senescence-associated (SA)-β-Gal and SAHF staining

Senescence was detected by using a β-Galactosidase Staining Kit from Cell Signaling Technology according to the manufacturer's instructions.

For SAHF detection, cells were fixed in 4% formaldehyde for 15 min at room temperature and per-meabilized with 0.25% Triton X-100 in PBS for 10 min at room temperature. Cells were then washed three times in PBS and incubated with DAPI (Invitrogen, Paisley, UK). Photomicrographs were taken using an Olympus BX41 microscope (Center Valley, PA).

### Western blot

Total protein was extracted from the treated tissues and cells using RIPA lysis buffer. After centrifugation at 10,000 g and 4°C for 30 minutes, 30 μg of protein extracts were boiled at 100°C in 1 × SDS loading buffer for 10 minutes, followed by processing using sodium dodecyl sulfate (SDS)-polyacrylamide gel electrophoresis (PAGE). Proteins were transferred to nitrocellulose membrane and blocked with 5% non-fat milk in Tris-buffered saline containing Tween-20 for 1 hour. The membrane was incubated with primary antibodies at 4°C overnight. Signals were detected with horseradish peroxidase (HRP)-conjugated goat anti-mouse or goat anti-rabbit IgG (1: 5000) (ThermoFisher Scientific, USA). Immunoreactive bands were visualized under enhanced chemiluminescence (ECL, Amersham Biosciences) and developed and quantified using an imaging system (Tanon, China).

### Immunoprecipitation

NP-40 (containing protease inhibitor cocktail and phenylmethanesulfonyl fluoride) was used to cleave the liver tissue, which was then centrifuged at 14,000 g at 4°C for 10 minutes. Then, 2 μg of the Pdia3 (Proteintech # 15967-1-AP) antibody was added to 50 μl of magnetic beads for 15 minutes at room temperature, and 1 mg of protein lysate was added to the beads to rotate overnight. A pre-cooled lysate was used to clean the beads three times, followed by the addition of 40 μl of 1X SDS loading buffer at 70°C for 5 minutes. Finally, the supernatant was then transferred.

### Chromatin immunoprecipitation

ChIP assays were performed as described previously (Chang & Guarente 2013). For ChIP, cell lysates were initially incubated with the anti-Clock antibody and antibody-coated magnetic beads (Cell Signaling, USA). Oligonucleotides used for the ChIP RT–PCR are listed in Table S1(Supporting information).

### Statistical analysis

All data were expressed as the mean ± S.E.M. A two-tailed Student's t-test was performed to determine the significance of the difference between the two groups. One-way analysis of variance (ANOVA) with Dunnett's post hoc test was used to compare more than two groups. P values < 0.05 were considered significant.

## SUPPLEMENTARY MATERIALS


