# Supplementary material for: Clock mediates liver senescence by controlling ER stress
Source: Aging (Albany NY). 2017 Dec 22;9(12):2647–65. doi: 10.18632/aging.101353 (PMC5764397; doi:10.18632/aging.101353)
Supplement: Supplementary file 1 [file aging-09-2647-s001.pdf]

SUPPLEMENTARY MATERIAL

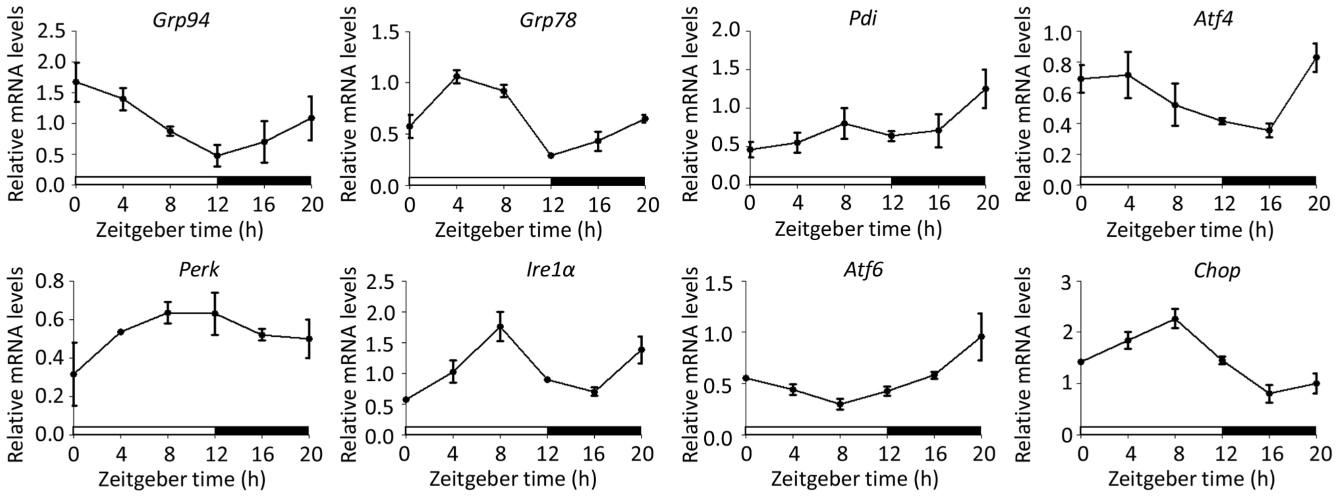

Supplementary Figure 1. UPR genes show circadian rhythm in the mouse liver.

Table S1. Mouse primers used for PCR analysis.

Gene		Primer Sequence	Ta(ºC)
Gapdh	Forward	AGGTCGGTGTGAACGGATTTG	60
	Reverse	TGTAGACCATGTAGTTGAGGTCA	
Grp78	Forward	AGTGGTGGCCACTAATGGAG	60
	Reverse	CAATCCTTGCTTGATGCTGA	
Perk	Forward	CGGAGACAGTGTTTGGCTTA	60
	Reverse	GCTTTTTCCCATCATTCTCG	
Pdi	Forward	GAGGACAACGTCCTGGTGTT	60
	Reverse	GCCTTCTGCCTTCAGTTTTG	

Pdia3	Forward	CGCCTCCGATGTGTTGGA	60
	Reverse	CAGTGCAATCCACCTTTGCTAA	
Pdia6	Forward	GGTGAGCTGCACCTTCTTTC	60
	Reverse	GCTGCTTTCTTCCATTCTGG	
Xbp-1(u)	Forward	TCCGCAGCACTCAGACTATGT	60
	Reverse	ATGCCCAAAAGGATATCAGACTC	
Xbp-1(s)	Forward	GAGTCCGCAGCAGGTG	60
	Reverse	GTGTCAGAGTCCATGGGA	
Grp94	Forward	CTGGGTCAAGCAGAAAGGAG	60
	Reverse	TCTCTGTTGCTTCCCGACTT	
Cat	Forward	ACATGGTCTGGGACTTCTGG	60
	Reverse	CAAGTTTTTGATGCCCTGGT	
Sod1	Forward	CCAGTGCAGGACCTCATTTT	60
	Reverse	TTGTTTCTCATGGACCACCA	
Sod2	Forward	CCGAGGAGAAGTACCACGAG	60
	Reverse	GCTTGATAGCCTCCAGCAAC	
Prdx1	Forward	TGCCAGATGGACAATTCAAA	60
	Reverse	GGTCCCAATCCTCCTTGTTT	

Prdx2	Forward	AGGACTTCCGAAAGCTAGGC	60
	Reverse	TTGACTGTGATCTGGCGAAG	
Gpx1	Forward	GTCCACCGTGTATGCCTTCT	60
	Reverse	TCTGCAGATCGTTCATCTCG	
Gpx2	Forward	GGCTTACATTGCCAAGTCGT	60
	Reverse	CTCCTGATGTCCGAACTGGT	
Gsr	Forward	CACGACCATGATTCCAGATG	60
	Reverse	CAGCATAGACGCCTTTGACA	
Gstk1	Forward	AATCGCTGGGATCATGAAAG	60
	Reverse	TTGCTCCATGCTTACAGTGG	
ChIP E-box	Forward	AGGCCAGAGGATGGAAAGTT	60
	Reverse	AGGGTGGGTAGCTGAGGATT	
